# Analysis of human brain tissue derived from DBS surgery

**DOI:** 10.1186/s40035-022-00297-y

**Published:** 2022-04-13

**Authors:** Salla M. Kangas, Jaakko Teppo, Maija J. Lahtinen, Anu Suoranta, Bishwa Ghimire, Pirkko Mattila, Johanna Uusimaa, Markku Varjosalo, Jani Katisko, Reetta Hinttala

**Affiliations:** 1 PEDEGO Research Unit, University of Oulu, Oulu, Finland; 2 Medical Research Center, Oulu University Hospital, University of Oulu, Oulu, Finland; 3 Biocenter Oulu, University of Oulu, Oulu, Finland; 4 Institute of Biotechnology, HiLIFE Helsinki Institute of Life Science, University of Helsinki, Helsinki, Finland; 5 Drug Research Program, Faculty of Pharmacy, University of Helsinki, Helsinki, Finland; 6 Neurosurgery, Research Unit of Clinical Neuroscience, Oulu University Hospital, University of Oulu, Oulu, Finland; 7 Oulu Research Group of Advanced Surgical Technologies and Physics (ORGASTP), Research Unit of Clinical Neuroscience, Oulu University Hospital, University of Oulu, Oulu, Finland; 8 Institute for Molecular Medicine Finland (FIMM), HiLIFE Helsinki Institute of Life Science, University of Helsinki, Helsinki, Finland; 9 Clinic for Children and Adolescents, Division of Pediatric Neurology, Oulu University Hospital, Oulu, Finland

**Keywords:** Deep brain stimulation, Movement disorders, Brain, Proteomics, Transcriptomics, RNA sequencing, LC–MS, Personalized medicine

## Abstract

**Background:**

Transcriptomic and proteomic profiling of human brain tissue is hindered by the availability of fresh samples from living patients. Postmortem samples usually represent the advanced disease stage of the patient. Furthermore, the postmortem interval can affect the transcriptomic and proteomic profiles. Therefore, fresh brain tissue samples from living patients represent a valuable resource of metabolically intact tissue. Implantation of deep brain stimulation (DBS) electrodes into the human brain is a neurosurgical treatment for, e.g., movement disorders. Here, we describe an improved approach to collecting brain tissues from surgical instruments used in implantation of DBS device for transcriptomics and proteomics analyses.

**Methods:**

Samples were extracted from guide tubes and recording electrodes used in routine DBS implantation procedure to treat patients with Parkinson’s disease, genetic dystonia and tremor. RNA sequencing was performed in tissues extracted from the recording microelectrodes and liquid chromatography-mass spectrometry (LC-MS) performed in tissues from guide tubes. To assess the performance of the current approach, the obtained datasets were compared with previously published datasets representing brain tissues.

**Results:**

Altogether, 32,034 RNA transcripts representing the unique Ensembl gene identifiers were detected from eight samples representing both hemispheres of four patients. By using  LC-MS, we identified 734 unique proteins from 31 samples collected from 14 patients. The datasets are available in the BioStudies database (accession number S-BSST667). Our results indicate that surgical instruments used in DBS installation retain brain material sufficient for protein and gene expression studies. Comparison with previously published datasets obtained with similar approach proved the robustness and reproducibility of the protocol.

**Conclusions:**

The instruments used during routine DBS surgery are a useful source for obtaining fresh brain tissues from living patients. This approach overcomes the issues that arise from using postmortem tissues, such as the effect of postmortem interval on transcriptomic and proteomic landscape of the brain, and can be used for studying molecular aspects of DBS-treatable diseases.

**Supplementary Information:**

The online version contains supplementary material available at 10.1186/s40035-022-00297-y.

## Background

Neurodegenerative diseases, especially Alzheimer’s disease and Parkinson’s disease (PD), are widely studied using postmortem brain tissues in the search for disease biomarkers and for understanding the molecular basis of the disease [1–5]. When using postmortem samples, the integrity of brain tissue is compromised due to the delay in collecting the samples, which may bias the results. Some proteins are more prone to degradation than others, and the observed outcome may depend on the postmortem interval [5, 6]. Likewise, RNA is rapidly degraded [7, 8], and fresh human brain transcriptome essentially differs from postmortem transcriptome [9]. Therefore, access to fresh brain tissues is critical for obtaining accurate information on brain-specific transcripts and transcriptome in vivo. Recently, approaches that utilize fresh, non-tumorous brain-derived samples from patients treated for various brain-affecting conditions have emerged. For example, brain biopsy samples were collected from patients suffering traumatic brain injury in conjunction with the insertion of an intracranial pressure-monitoring device during corticotomy [10].

Deep brain stimulation (DBS) is a neurosurgical treatment for advanced and medically refractory movement disorders, such as PD, essential tremor and dystonia. In addition, pain, epilepsy and psychiatric disorders are increasingly treated with DBS [11]. During DBS operation, intracranial electrodes are targeted into specific locations in the deep brain structures bilaterally. The intracranial leads are connected to an external impulse generator through extension leads. The DBS device stimulates deep brain structures with a low-level electrical current that alleviates patients’ symptoms in a reversible manner. Zaccaria et al*.* have previously collected brain-derived material during DBS surgery from several individual patients with PD for proteome and transcriptome analysis [12]. Their approach included an additional step during the surgery, where a blunt stylet was inserted into the brain tissue to collect the material for analysis.

**Fig. 1:**
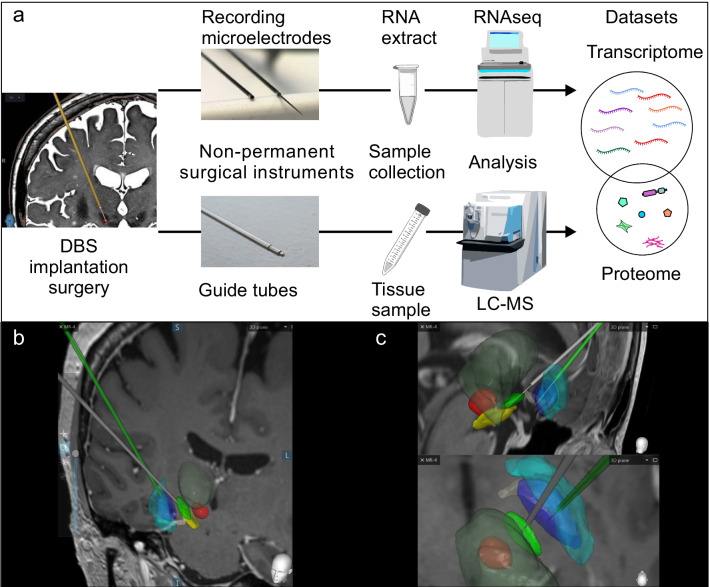
Workflow to collect fresh brain material during DBS surgery. **a** DBS leads were implanted to treat movement disorders in neurosurgical operation at Operative Care Unit, Oulu University Hospital. The tissue samples for LC–MS were collected from the guide tubes, which protruded through the brain tissue to reach the target area and therefore contained tissue material from different brain regions (e.g., cortex and white matter). The samples for RNA sequencing were collected from the recording microelectrodes targeted to the subthalamic nucleus (STN) or globus pallidus interna (GPi). **b** An image showing how the guide tubes (grey and green thick lines) passed through brain tissue, with the most distal end 10 mm from the planned target. **c** In contrast, the microelectrodes (thin grey lines) travelled inside the guide tube, and they touched brain tissue only in the STN (green area) and GPi (blue area). To help with anatomical orientation, other brain structures are marked, including the thalamus (dark transparent green), substantia nigra (yellow), red nucleus (red), ansa lenticularis (dark white) and globus pallidus externa (transparent turquoise)

In this study, we set out to assess whether the surgical, non-permanent instruments used in the standard DBS implantation procedure as followed at the Operative Care Unit at Oulu University Hospital [13] contain enough hemisphere-specific brain-derived material from individual patients for RNA sequencing (RNA-seq) and liquid chromatography-mass spectrometry (LC–MS) (Fig. 1a).

## Methods

### Patients

DBS lead implantation was performed during neurosurgical operations at the Operative Care Unit, Oulu University Hospital, Finland, between October 2017 and June 2019. The indications for DBS treatment were PD (*n* = 13), genetic dystonia (*n* = 3) and tremor (*n* = 1). Guide tubes and microelectrodes were used during the standard DBS implantation procedure (Fig. 1a). The samples were obtained from the recording microelectrodes collected from four patients (eight samples, Table 1) for RNA sequencing (RNA-seq) and the guide tubes from the procedures of 14 patients (31 samples, Table 2) for LC–MS. The samples were collected separately from the left and the right hemispheres. The extracted RNA originated from the subthalamic nucleus (STN) of two PD patients and from the globus pallidus interna (GPi) of two dystonia patients (Fig. 1c). The detailed information on the patients and the samples, and total numbers of transcripts identified by RNA-seq in each sample are listed in Table 1.

**Table 1:** Information on patients and collected samples for RNA-seq analysis

Patient	Sex	Age	Movement disorder	Target area	Sample ID	Brain hemisphere	Number of transcripts identified by RNAseq
1^a^	M	8	Dystonia	GPi	DYT1R_C	Right	19,343
					DYT1L_C	Left	23,817
15	M	67	PD	STN	PD12L	Left	22,411
					PD12R	Right	21,522
16	F	60	Dystonia	GPi	DYT3L	Left	11,861
					DYT3R	Right	20,440
17	F	61	PD	STN	PD13R	Right	21,880
					PD13L	Left	17,311

M, male; F, female; PD, Parkinson’s disease; DYT, dystonia; GPi, globus pallidus interna; STN, subthalamic nucleus; R, right hemisphere; L, left hemisphere

^a^Samples of the patient 1 were collected from the DBS reimplantation procedure

**Table 2:** Information on patients and collected samples for LC–MS analysis

Patient	Sex	Age (years)	Movement disorder	DBS target area	Sample ID	Brain hemisphere	Visible blood	Total protein (μg)	Number of proteins identified
1^a^	M	6	Dystonia	GPi	DYT1L_A	Left	Yes	453.11	181
					DYT1R_A	Right	No	4.28	298
1^a^	M	7	Dystonia	GPi	DYT1L_B	Left	Yes	50.52	163
					DYT1R_B	Right	Yes	48.88	287
2	M	54	Tremor	VIM	TRE1R	Right	No	8.51	217
					TRE1L	Left	No	NA	190
3^b^	M	67	PD	STN	PD1L	Left	No	NA	165
					PD1R1	Right	No	14.25	375
					PD1R2	Right	No	NA	218
4^c^	F	58	PD	STN	PD2R	Right	Yes	82.63	199
					PD2L	Left	No	6.03	319
5	M	58	PD	STN	PD3L	Left	Yes	86.80	153
					PD3R	Right	Yes	148.61	252
6^c^	M	62	PD	STN	PD4L	Left	Yes	63.82	211
					PD4R	Right	Yes	93.01	175
7	M	59	PD	STN	PD5R	Right	No	2.36	244
					PD5L	Left	Yes	87.60	262
8	F	67	PD	STN	PD6R	Right	No	NA	292
					PD6L	Left	Yes	20.07	161
9	M	59	PD	STN	PD7L	Left	No	3.87	136
					PD7R	Right	No	NA	150
10	M	52	PD	STN	PD8R	Right	No	NA	193
					PD8L	Left	No	NA	67
11	F	63	Dystonia	GPi	DYT2L	Left	No	NA	120
					DYT2R	Right	No	NA	200
12	M	59	PD	STN	PD9L	Left	No	NA	178
					PD9R	Right	Yes	9.24	261
13	F	66	PD	STN	PD10L	Left	Yes	14.79	223
					PD10R	Right	No	2.51	211
14	F	62	PD	STN	PD11R	Right	No	NA	169
					PD11L	Left	No	NA	204

M, male; F, female; PD, Parkinson’s disease; DYT, dystonia; TRE, tremor; GPi, globus pallidus interna; STN, subthalamic nucleus; VIM, ventral intermediate nucleus of the thalamus; R, right hemisphere; L, left hemisphere; NA, not available

^a^From the Patient 1, two sets of LC–MS samples were obtained from two separate surgical procedures. The first set of samples (DYT1L_A and DYT1R_A) was collected from the guide tubes during the first DBS implantation procedure. The second set of samples (DYT1L_B and DYT1R_B) was collected from the revised DBS leads during the revision surgery, which was performed due to technical failure

^b^Of the three samples from Patient 3, two (PD1R1 and PD1R2) were from the right hemisphere

^c^The LC–MS samples from Patients 4 and 6 (sample codes PD2 and PD4, respectively) were obtained during re-implantation to resume DBS treatment after removal of the previous DBS leads due to technical failure

For tissues from the guide tubes for proteomics analysis, the intracranial leads were targeted into the STN of the PD patients (23 samples from 11 patients), GPi of the dystonia patients (6 samples from 2 patients), or ventral intermediate nucleus of the thalamus (VIM) of the tremor patient (2 samples from 1 patient). The information on patients and samples, presence of visible blood in the samples, total protein amounts and numbers of proteins identified in each sample are listed in Table 2.

### DBS implantation procedure

The surgical procedure for DBS implantation was carried out according to the standard protocol in our institute, as described in detail by Lahtinen et al*.* [13]. The patient-specific targeting of intracranial electrodes was planned on brain magnetic resonance imaging (MRI) images and adjusted with intraoperative clinical testing and microelectrode registration (MER) during neurosurgical operation if the patient was awake. The guide tubes (Universal Guide Tube, Elekta, Stockholm, Sweden) and the recording microelectrodes (Leadpoint, Alpine Biomed, Skovlunde, Denmark) used during the implantation procedure were collected and used for sample acquisition for proteomics and transcriptomics, respectively. The location of the intracranial electrodes is most commonly in the deep basal nuclei, and the most common trajectory to the target area is through the posterior parts of the frontal lobes (Fig. 1b, c).

### RNA extraction for RNA-seq

After removal from the brain, the recording microelectrode was placed on ice and taken to a research laboratory, where it was immediately immersed in 700 µl of QIAzol Lysis Reagent (QIAGEN, Hilden, Germany) at room temperature and triturated by using a lead as the piston. The microelectrode was kept in QIAzol Lysis Reagent for about 10 min and triturated once more before discarding the electrode. The sample was briefly vortexed (2–3 s) and stored at − 80 °C. RNA-seq was performed by the Sequencing Unit of the Institute for Molecular Medicine Finland FIMM Technology Centre, University of Helsinki.

### RNA-seq

Total RNA was extracted with a Qiagen miRNeasy micro kit (QIAGEN, Hilden, Germany), according to the kit handbook. The quality and quantity of the extracted RNA samples were analyzed with a 2100 Bioanalyzer using an RNA 6000 Pico Kit (Agilent, Santa Clara, CA). Paired-end cDNA libraries were prepared from 0.2 ng of extracted RNA, with 11 cycles of amplification using a SMART-Seq v4 Ultra Low Input RNA Kit, according to the manufacturer’s user manual (Takara Bio USA, Inc. Mountain View, CA). One hundred pg of amplified cDNA was tagmented and indexed for sequencing using a Nextera XT DNA Library Prep Kit (Illumina, San Diego, CA). LabChip GX Touch HT High Sensitivity assay (PerkinElmer, USA) was used for quality measurement and quantification of the purified dual-indexed libraries for equimolar pooling. The sequencing of the pooled samples was performed with an Illumina NovaSeq 6000 System (Illumina, San Diego, CA). The read length for the paired-end run was 2 × 101 bp, and the target coverage was 15 M reads for each library.

### RNA-seq data analysis

**Fig. 2:**
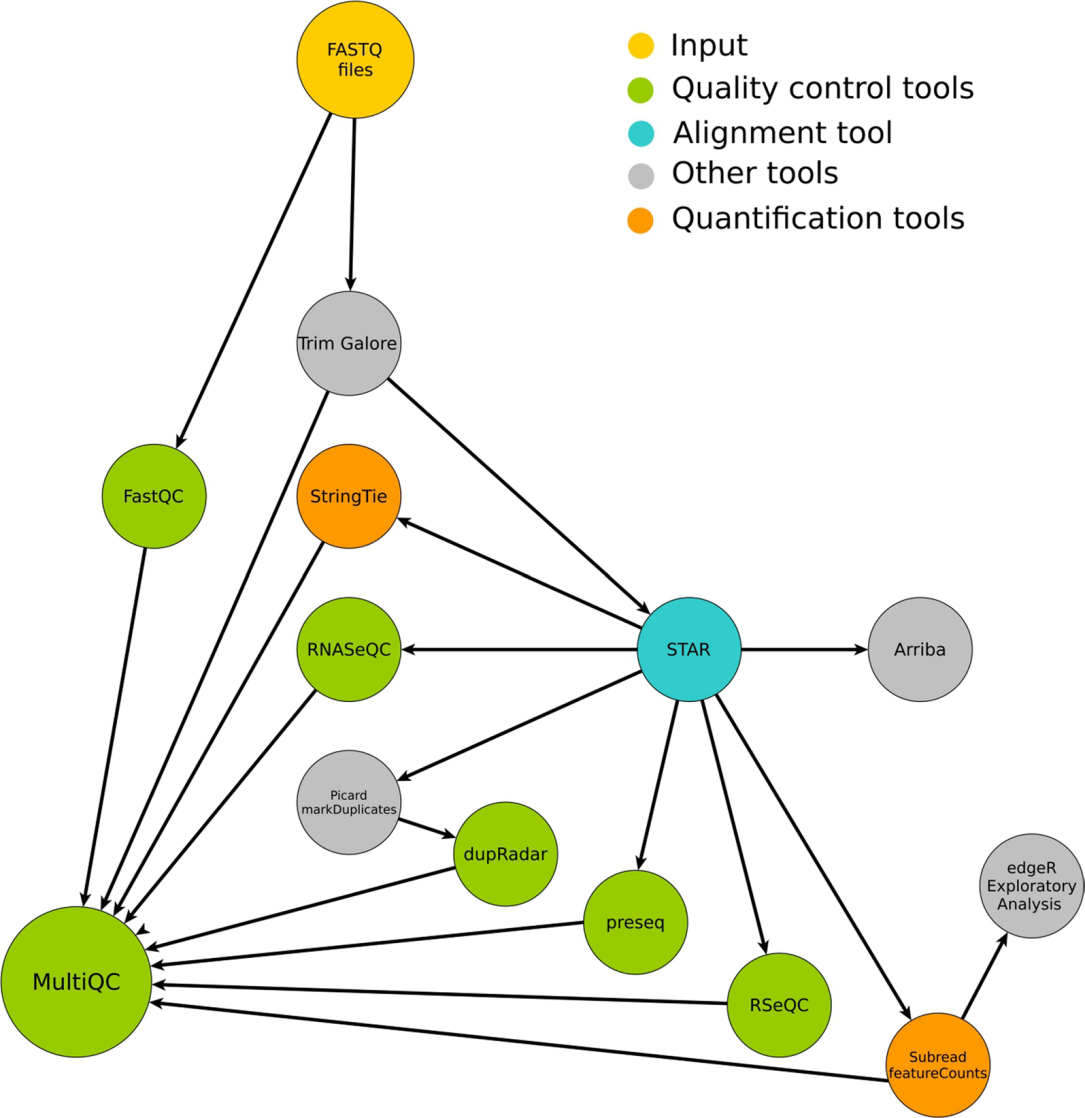
FIMM-RNAseq data analysis pipeline. FIMM-RNAseq incorporates quality control tools, such as FastQC and the pre-processing tool Trimgalore. It aligns RNA-seq reads using a STAR [14] aligner and performs gene quantification and transcript assembly using Subread [15] and StringTie [16], respectively. Extensive RNA-seq quality matrices are generated using RNASeQC [17], RseQC [18], dupRadar [19] and Preseq [20, 21]. An aggregated report from the major analysis steps is generated using MultiQC [22]. Exploratory data analysis is performed using R and edgeR [23]. As an optional component, the pipeline has the gene-fusion prediction tool Arriba [24]

The RNA-seq datasets were analyzed using FIMM-RNAseq data analysis pipeline Version v2.0.1. (Fig. 2). The pipeline is implemented in Nextflow [25]⁠. Nextflow allows the portability and scalability of the pipeline and supports major cloud computing and batch processing technologies. More importantly, the pipeline allows the reproducibility of the results by using a version-labeled set of software dependencies, a Conda environment. The Conda environment can be created manually, or created by Nextflow. Alternatively, a readily available Docker image containing all software dependencies can be used to run the pipeline in a containerized computing environment, such as Docker and Singularity. Source code and a comprehensive user’s manual of the pipeline are available at https://version.helsinki.fi/fimm/fimm-rnaseq

### Brain tissue sample collection for LC–MS

The guide tubes were transported from the operation room to the laboratory on ice, immediately after removal from the brain, and the samples were prepared for cryopreservation within one hour, as described below. The instruments from different hemispheres of each patient were handled individually.

The guide tubes were rinsed from inside with 10 ml of ice-cold phosphate-buffered saline (Sigma-Aldrich, St. Louis, MO) using a 27-gauge needle and syringe. The suspension was collected into a 15-ml conical tube on ice. The tissue was pelleted by centrifugation at 400 g for 15 min at 4 °C. After centrifugation, the supernatant was removed carefully, and the pellet was flash frozen in liquid nitrogen. The samples were stored at − 70 °C until analysis.

### Sample preparation and LC–MS analysis

The cells were lysed, and the proteins denatured by adding 200 μl of 8 mol/l urea (Sigma-Aldrich, St. Louis, MO), followed by 15-min sonication. Insoluble cell debris was removed via two rounds of centrifugation (15 min, 20,817 g, 22 °C). Total protein content was measured with BCA assay (Thermo Scientific, Waltham, MA), the results of which are shown in Table 2.

Disulfide bonds were reduced with dithiothreitol (final concentration 5 mmol/l; Sigma-Aldrich, St. Louis, MO), and the cysteine residues were carbamidomethylated with iodoacetamide (final concentration 15 mmol/l; Sigma-Aldrich, St. Louis, MO), after which a pooled quality control (QC) sample was created by taking 31 μl of each sample and combining them. The proteins were digested with 2.5 μg of sequencing-grade modified trypsin (Promega, Madison, WI). The resulting peptides were purified with C18 MicroSpin columns (The Nest Group, Inc., Ipswich, MA); for samples with > 60 μg of total protein, only 60 μg of total digested protein was taken for C18 purification, whereas for samples with < 60 μg of total protein, all of the sample was used. After C18 purification, the samples were evaporated to dryness with a vacuum centrifuge and stored at − 20 °C.

Prior to LC–MS analysis, the samples were resolubilized with 15-min sonication in 30 μl of 1% acetonitrile + 0.1% trifluoroacetic acid in LC–MS grade water (all from VWR, Radnor, PA). The injection volume (between 2 and 10 μl) was determined based on the amount of total protein in the sample. The sample was injected into the LC–MS, separated with EASY-nLC 1000 (Thermo Scientific, Waltham, MA) using a 120-min linear gradient and detected with Orbitrap Elite MS (Thermo Scientific) using top20 data-dependent acquisition, in which the 20 most intense ions from each MS1 full scan were fragmented and analyzed in MS2. Pooled QC samples were analyzed at the beginning and the end of the run sequence, but they were removed from the final data analysis.

Protein identification and quantification were performed with Andromeda and MaxQuant [26, 27] with the standard settings and using a reviewed *Homo sapiens* UniProtKB/Swiss-Prot proteome (20,431 entries, downloaded on 2019-08-30; The Uniprot Consortium [28]). In addition, label-free quantification (LFQ) was enabled, and identification FDR < 0.01 filtering was applied on both peptide and protein levels. The LFQ intensity was used as an estimate of protein abundance without further normalization. From the output, we filtered decoy hits, proteins flagged as potential contaminants (but not serum albumin) and proteins identified with a modification site only. The LFQ intensities of all quantified proteins in all samples are presented in the Additional file 1.

To account for the variable amounts of blood in the samples, the correlations of each protein’s LFQ intensity with those of serum albumin and hemoglobin subunit alpha were calculated, but no filtering based on these correlations was applied. The correlations are listed in the Additional file 1.

### Bioinformatics

Hierarchical clustering and principal component analysis (PCA) were applied to the data in order to see whether there are any obvious patterns of separation between the samples. For the proteomics dataset, the data were centered and scaled to zero mean and unit variance before applying hierarchical clustering (Euclidean distance metric, complete linkage) or PCA. For RNA-seq dataset, PCA and hierarchical clustering (Correlation distance metric, complete linkage) were performed using normalized feature counts.

Allen Brain Atlas Adult Human Brain Tissue Gene Expression Profiles [29] for the STN and GPi were downloaded from https://maayanlab.cloud/Harmonizome/dataset/Allen+Brain+Atlas+Adult+Human+Brain+Tissue+Gene+Expression+Profiles. The reference lists were formed by including all the upregulated genes from both hemispheres of the specific anatomical structure, STN or GPi, to the same list. The g:Convert tool of the g:Profiler [30] web server was used to convert the identifiers to the same namespace (ENSG_ID).

The list of the gene transcripts detected in human basal ganglia (*n* = 14,736) was from Human Protein Atlas (https://www.proteinatlas.org/humanproteome/brain) [31]. The list of Ensembl identifiers was used for comparison with our RNA-seq dataset.

The brain region- and hemisphere-specific postmortem datasets were from the publication by Biswas et al*.* [32]. For enrichment analysis, a proteomics dataset that included proteins expressed in the frontal cortex and the frontal white matter was generated. The datasets used for the enrichment analyses are included in Additional file 2.

To study clustering of the RNA-seq samples according to specific transcripts, the list of the 13 proteins (FKBP4, GMFB, GNB2, HNRNPA1, HSPA6, PA2G4, PEA15, PYGM, SDHA, SELENBP1, SLC9A3R1, SNX3 and UBA52) found to be differentially expressed in the basal ganglia regions were picked from the publication by Biswas et al*.* [32]. For the hierarchical clustering and expression heatmap, transcription of each target gene per million expression levels was analyzed. To be able to compare the relative expression levels between the two hemispheres of each individual, the expression level of each of the analyzed genes was normalized across the two hemispheres as follows: (E(gene)^L^ − E(gene)^R^)/(E(gene)^L^ + E(gene)^R^) for the left hemisphere and (E(gene)^R^ − E(gene)^L^)/(E(gene)^L^ + E(gene)^R^) for the right hemisphere, where E(gene) is gene-specific expression level and superscripts L and R indicate left and right hemispheres, respectively. The analysis was done with OriginPro 2022 (version 9.9.0.225) Heat Map with the Dendrogram tool, using hierarchical clustering by furthest neighbour (complete linkage) and Pearson correlation.

The SynGO portal [33] was used to analyze the enriched terms in the RNA-seq dataset. The used dataset was prepared for analysis by using the gene list that contained the overlapping genes among all RNA-seq samples (*n* = 9901). To make a list of the top 20% of expressed genes among this common gene set, the expression levels of individual genes were normalized against the total expression level of the sample. The average value of normalized expression level was used to rank the genes from high to low, and the top 20% (*n* = 1980) identifiers were used for SynGO analysis. A list of the ranked genes and original SynGO results are shown in Additional file 3.

Gene ontology (GO) [34, 35] and KEGG pathway [36] enrichment analysis using the g:Profiler g:GOSt tool [30] (https://biit.cs.ut.ee/gprofiler/gost, version e104_eg51_p15_3922dba, database updated on 07/05/2021) was performed for the list of all identified proteins across all samples using Uniprot identifiers, with ambiguous query genes being excluded. The complete lists of all enriched GO terms and KEGG pathways (g:SCS algorithm for multiple testing correction, significance threshold being < 0.05) are included in Additional file 2.

BioVenn [37] was used to draft area-proportional Venn diagrams. InteractiVenn was used to draw other Venn diagrams [38].

## Results

### RNA-seq analysis produces tissue-specific data

**Fig. 3:**
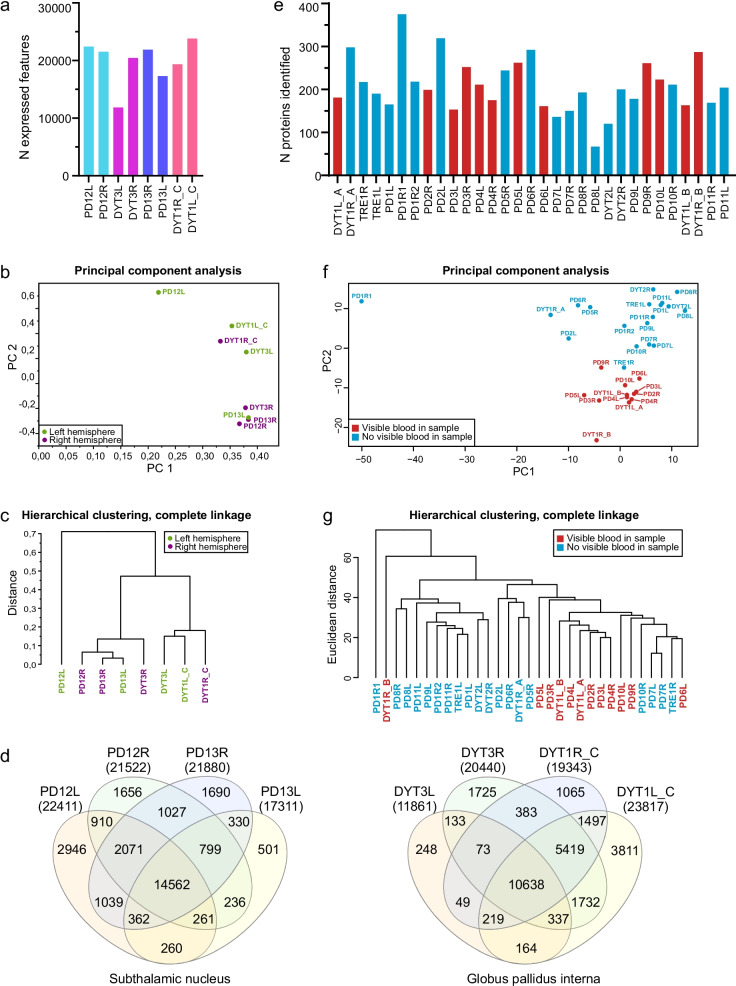
Features of proteomics and transcriptomics datasets obtained from the RNA sequencing and LC–MS analyses of the patient-derived brain tissues. The sample encoding indicates the patients’ disorders as follows: Parkinson’s disease (PD, *n* = 13), genetic dystonia (DYT, *n* = 3) and tremor (TRE, *n* = 1). **a** The number of expressed genes in each sample. **b** Principal component analysis plot of the gene expression data. **c** Hierarchical clustering, colored based on the hemisphere, shows that the samples tended to cluster according to the DBS target region. **d** Venn diagrams showing the number of common genes identified in the samples from the subthalamic nucleus (STN) and the globus pallidus interna (GPi). **e** The number of identified proteins in each sample, colored based on whether blood was visible in the sample. No statistical difference was observed in the number of proteins identified (*P* = 0.51, *t*-test). **f** Principal component analysis plot of the proteomic data. **g** Hierarchical clustering, colored based on whether blood was visible in the sample, shows that samples with visible blood tended to cluster

Transcriptomic analysis was focused on the STN and GPi regions, which are specific targets of DBS in treating patients with movement disorders (Fig. 1c). Samples for RNA-seq were collected separately from the recording microelectrodes targeted to both hemispheres of four patients (Table 1), of whom two had PD with STN as the target area and two had genetic dystonia with GPi as the target area. The number of identified genes expressed in the eight samples varied from 11,861 to 23,817 (Fig. 3a), of which 32,034 genes were unique across all the samples (Additional file 4). The sample DYT3L had notably lower number of reads compared to other samples, which might be a result of RNA degradation in this specific sample. PCA (Fig. 3b) and hierarchical clustering (Fig. 3c) were performed to evaluate the clustering of the samples according to their whole transcriptome profiles. In this case, there was no clear trend in clustering according to hemisphere, but the samples tend to cluster according to anatomical target region, STN or GPi. In total, 14,562 genes identified were shared between all samples from the STN, and 10,638 genes identified were shared between all samples from the GPi (Fig. 3d). Also, 9901 genes were commonly detected in all samples from both STN and GPi regions.

### LC–MS can be used to analyze the brain hemisphere-specific tissue samples attached to the guide tubes

Proteomics analysis was carried out on the tissue material attached to the guide tubes that were used to reach the microelectrode target region (Fig. 1b). After confirming by immunoblotting that the brain-specific proteins were detectable in the tissue material collected from guide tubes (Additional file 5), we proceeded with LC–MS analysis. The LC–MS analysis identified 734 unique proteins in 31 samples from 14 patients (Table 2). Eighteen of these proteins (seven being abundant in blood) were present and quantified in all samples (Additional file 1). Based on visual inspection, the samples contained variable amounts of blood, which, however, did not influence the overall number of proteins identified in those specific samples (Fig. 3e). Clustering of samples according to the blood observed in them was evident (Fig. 3f, g). When we analyzed the identified protein datasets using G:profiler G:OST tool [30], we found that the enriched GO terms reflected the brain tissue well, and blood, which was observed in some of the samples, was not over-represented among these terms (Fig. 5b, Additional file 2). When the transcriptomics dataset was mapped to Uniprot identifiers using g:Profiler [30], there were 686 identifiers overlapping between the transcriptomics dataset and proteomics dataset, which covered 93.5% of all identified proteins.

### Comparison between the current approach and the previously published method by Zaccaria et al.

Zaccaria et al*.* have previously utilized DBS surgery to obtain brain-derived material from PD patients, which they termed “brain tissue imprints” (BTIs), for proteome and transcriptome analysis [12]. To our knowledge, this is the only published method that resembles ours; however, there are substantial differences in procedure. We compared our approach and results to the approach published by Zaccaria et al*.* (Table 3) [12].

**Table 3:** Comparison of the two approaches to collect and analyze samples obtained during DBS surgical procedure

	Current paper	BTI method [12]
Sample collection protocol	The brain tissue attached to the guide tube and microelectrode during standard DBS implantation procedure was used for sample preparation	A blunt stylet was inserted through the guide tube into the brain tissue during DBS implantation procedure for one minute to obtain material for analyses
Sample usage	Both RNA-seq and LC–MS analyses can be carried out from the same individual patients and their separate brain hemispheres (if patient is awake during the procedure)	Tissue sample was used either for RNA microarray analysis or pooled for Nano-LC–MS/MS. Samples were also alternatively used for immunocytochemistry or scanning electron microscopy
Transcriptomics	The tissue material was collected from the recording microelectrode targeted to the specific well-defined area in the deep brain region and was prepared for RNA-seq	RNA microarray for the tissue sample attached to the blunt stylet was carried out after application of double amplification protocol
Proteomics	No pooling of samples. Hemisphere-specific LC–MS data were obtained from the tissue collected from the guide tube	Six samples from different patients and brain hemispheres were pooled for in-gel fractionation and subsequent MS analysis

Zaccaria et al*.* collected 19 samples from 12 patients as follows: after determining the DBS target area via MER, a blunt stylet, as an additional sample collection step, was inserted through the guide tube into the brain for one minute to obtain material for analyses. The material attached to the stylet was then used for proteomics, electron microscopy, immunohistochemistry and immunofluorescence or RNA microarray analysis. In our protocol, no alterations or additional steps were introduced to the standard DBS procedure; instead, we collected the brain tissue material that had attached to the guide tubes and recording microelectrodes during the normal surgical procedure. Because our DBS implantation surgery followed standard procedure, we were able to collect samples systematically from both hemispheres of each patient, whereas the protocol used by Zaccaria et al*.* had technical constraints that allowed sample collection procedure from both hemispheres only occasionally. An essential difference in the transcriptomics data output was that Zaccaria et al*.* performed RNA microarray analysis whereas we performed RNA sequencing. RNA microarray analysis profiles predefined transcripts through hybridization while RNA sequencing is quantitative and covers the whole transcriptome and therefore it can be used to detect different not-predefined transcripts. During proteomics analysis, Zaccaria et al*.* pooled multiple samples, while we analyzed hemisphere-specific individual samples.

### Comparison of the datasets to the previously published data

**Fig. 4:**
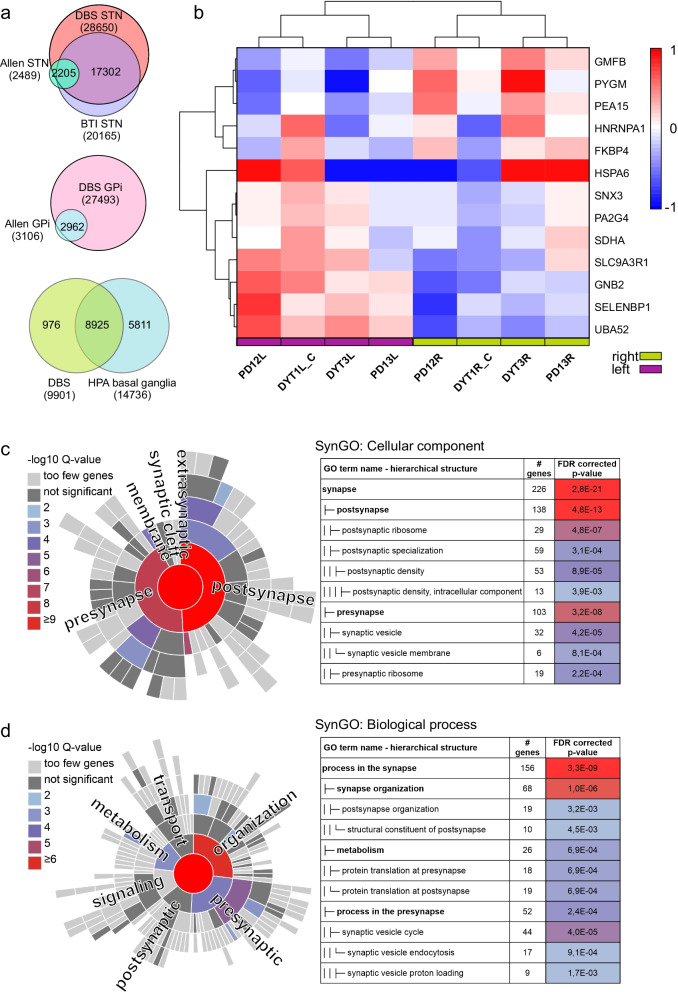
Comparison of the RNA-seq dataset to previously published data and SynGO enrichment analyses. **a** Upper panels: there was a substantial overlap in subthalamic nucleus (STN) and globus pallidus interna (GPi)-specific terms between our transcriptomics datasets and the anatomically specific expression datasets in Allen Brain Atlas [29]. Our STN data also had 86% overlap with the BTI dataset. Bottom panel: 90% of transcripts common to all samples in our RNA-seq dataset were also found to be common with Human Protein Atlas (HPA) basal ganglia-specific expression dataset [31]. **b** The gene expression patterns of 13 proteins, that were identified by Biswas et al*.* [32] to be differentially expressed in the basal ganglia between the left and right hemispheres, also clustered according to hemisphere in the clustering analysis based on our RNA-seq data. We tested the top 20% expressed RNA-seq identifiers common to all analyzed samples (*n* = 1980) using the SynGO Knowledge base gene set enrichment tool [33]. **c** Ten terms in the cellular component category and **d** 11 terms in the biological process category were significantly enriched at 1% FDR (testing terms with at least three matching input genes)

The datasets achieved by our method were compared to the datasets obtained using the BTI approach described by Zaccaria et al*.* [12] and postmortem samples from specific brain regions [31, 32] (Fig. 4a). Zaccaria et al*.* produced three RNA microarray datasets from the STN of three patients, which detected 35,701, 29,842 and 27,350 unique microarray probe identifiers. We converted the probe identifiers from the BTI RNA microarray dataset with g:Profiler [30] into 20,165 unique Ensembl gene (ENSG) identifiers to allow comparison with our dataset. Ultimately, 17,302 unique identifiers (86%) were common to our STN-specific RNA-seq and BTI microarray datasets. We also compared our STN- and GPi-specific RNA-seq datasets to brain region-specific expression datasets representing up-regulated genes in the STN and GPi (Allen Brain Atlas) [29] and found that a substantial majority (85% and 95%, respectively) of the upregulated genes were present in our datasets. Our expression data originated from the STN and GPi, which are subregions of basal ganglia. Therefore, we compared the 9901 commonly expressed genes in our samples to the basal ganglia-specific genes listed in Human Protein Atlas [31] and found an overlap of ~ 90% of our dataset.

Biswas et al*.* studied, for the first time, brain hemisphere-specific proteome in several brain anatomical regions [32] from one individual and they identified 13 differentially expressed proteins between right and left basal ganglia. Interestingly, when we did the clustering analysis based on the RNA-seq data of these 13 genes identified by Biswas et al*.,* our samples also clustered according to hemisphere (Fig. 4b).

SynGO is a knowledgebase that focuses on synapse-specific ontologies, and its annotations are based on published, expert-curated evidence [33]. Koopmans et al. have shown that synaptic genes are exceptionally well conserved and less tolerant of mutations than other genes [33]. They conclude that many SynGO terms are overrepresented for genes that have variants associated with brain disease. By using the SynGO analysis tool, we could identify several terms enriched among the 20% top of expressed genes (Fig. 4c, d, Additional file 3), and 68% (754/1112) of SynGO annotated genes were found in our RNA-seq dataset that contained 9901 overlapping genes among the eight samples. This indicates that the RNA-seq data obtained from the tissue attached to the recording microelectrodes during the DBS implantation procedure are a potentially useful resource for studying brain disorders and the brain-specific transcriptome landscape, such as brain-specific transcript isoforms in vivo.

**Fig. 5:**
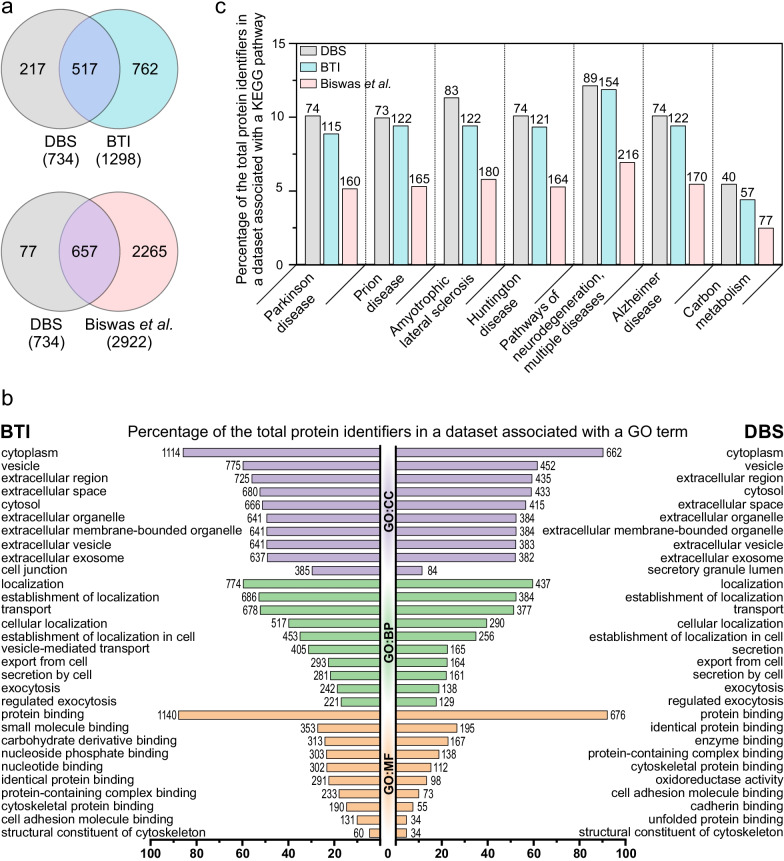
Comparison of the proteomics dataset to other published data and enrichment analyses. To obtain an overview of the type of proteins identified, GO and KEGG pathway enrichment analysis using g:GOst analysis tool [30] was performed to the list of all identified proteins across all the samples. We also performed the same analysis to the BTI dataset [12] and the region-specific dataset [32] to compare the outcomes of different approaches. **a** The DBS dataset of all unique protein identifiers was compared to the BTI proteomics dataset [12] and the region-specific dataset representing frontal cortex and frontal white matter by Biswas et al*.* [32]. Out of 734 identifiers, 517 (70%) were in common with the BTI dataset and 657 (90%) were in common with the frontal cortex and frontal white matter region-specific dataset. **b** The top 10 most enriched terms in each GO category (Cellular component, CC; Biological process, BP; Molecular function, MF) showed that the DBS dataset and the BTI dataset shared many top terms with similar enrichment pattern. **c** The number and fraction of identifiers belonging to top enriched KEGG pathways in our DBS dataset were compared with the same enriched pathways identified in the BTI [12] dataset and the frontal cortex and white matter region-specific dataset [32]. Most of the top enriched pathways were common to all three datasets. In **b** and **c**, the length of the column indicates the percentage of the features identified in each dataset falling into each category and the numerical value indicates the number of features identified as a part of each feature

The BTI protocol for sample acquisition led to the identification of 1298 unique proteins from the pooled samples using Nano-LC–MS/MS [12]. We compared the list of our identified proteins to the BTI proteomics dataset and found that 70% of the proteins in our dataset overlapped with the BTI proteomics dataset (Fig. 5a). We also compared our dataset to the proteomics data by Biswas et al*.* [32] by compiling a dataset that included the identified proteins in the frontal cortex and frontal white matter regions. Frontal cortex and frontal white matter are two main brain regions that are penetrated by the guide tube during the DBS surgery to reach the target of the electrode (Fig. 1b). Around 90% of the proteins in our dataset overlapped with the dataset by Biswas et al*.* (Fig. 5a).

The GO enrichment analysis of our current and the BTI proteomics datasets via the g:Profiler g:GOst tool revealed that the two datasets had many similar GO term profiles in their top 10 enriched terms (Fig. 5b). We also ran similar analysis for the dataset by Biswas et al*.* [32] in which we focused on the proteins identified in the frontal cortex and cortical white matter, brain regions penetrated by the guide tube. The distribution of the GO terms and KEGG pathways in the dataset by Biswas et al*.* was very similar with the DBS and BTI datasets (Additional file 2). The top seven enriched KEGG pathways in our DBS dataset were used for more detailed comparison (Fig. 5c). Fractions of the proteins connected to each term were very similar across the datasets and the KEGG pathways related to neurological diseases were the most enriched in all three cases (Fig. 5c, Additional file 2).

## Discussion

Previously published proteomics and transcriptomics datasets for different human brain areas were most often based on postmortem material because brain biopsies from living patients are hardly achievable. When using postmortem samples, the integrity of brain tissue is compromised due to the delay in collecting the samples, which may bias the results. Dachet et al*.* showed that, during the postmortem interval, within few hours, neuronal gene expression, especially expression of brain activity-dependent genes, declines rapidly, while astroglial and microglial gene expression increases reciprocally [9]. In turn, most of the housekeeping genes, which are frequently used for normalization of expression levels, are very stable. Also, reduced diversity in the complexity of differentially spliced transcripts of RBFOX1, which otherwise has ultra-complex splicing patterns, was demonstrated [9]. This results in transcriptomics and proteomics data that do not correspond to the normal expression landscape of functional brain tissues. Specific transcripts and posttranslational modifications may reflect the disease stage and thus function as disease (stage) biomarkers. Using fresh material that is processed rapidly within a known time window reduces the technical variation caused by postmortem changes. Biopsies from brain tumors, such as gliomas, are one source of fresh brain-derived tissue that has been utilized quite widely in various omics approaches during the past years [39, 40], even though they represent the neoplastic phenotype, which does not correspond to normal brain tissue. In this study, we collected intact brain tissues of living patients from the surgical instruments used in DBS surgery, for subsequent proteomics and transcriptomics analyses. The instruments were obtained immediately after their use in the surgical operation to ensure the integrity of the brain tissue, and the samples were rapidly frozen to avoid degradation. At the same time, while our current approach overcomes the problem of brain tissue change during the postmortem interval, it has a limitation when it comes to neurologically intact control group, because it is impossible to acquire similar tissue biopsies from healthy individuals due to ethical reasons. Compared to postmortem samples, DBS implantation-derived samples present earlier time points in the disease course and phenotype, which helps in understanding the changes that occur at defined clinical stages during the development of neurological symptoms.

Even though acquisition of healthy control samples using this method is not possible, a comparison of the molecular signatures between different diseases and different stages of disease progression allows the identification of common brain-specific proteoforms and transcripts, as well as novel disease biomarkers, for further studies. If a neurosurgical operation is performed on a conscious patient to perform MER and thus adjust the target region, at the same time, samples for transcriptomics can also be collected from the microelectrodes, representing a very defined brain target area. In contrast to the highly region-specific transcriptomics analysis, our proteomics analysis provides data from a cross-section of the brain, containing an expression profile from a mixture of cell types from different brain layers e.g., cortex and white matter. Furthermore, our results indicate that the approach is applicable in studying brain disorders at the individual hemisphere level. Recently, Biswas et al*.* published a postmortem proteomics study where they compared the brain region- and hemisphere-specific expression patterns in an individual donor [32]. They suggest that there are probably hemisphere-specific differences in the expression patterns in the anatomical regions, since the different functional activities are localized to different hemispheres. Also, some brain-affecting diseases, such as PD, start by asymmetric degeneration in the brain [41], which emphasizes the importance of access to hemisphere-specific molecular information. We noticed brain region-specific clustering of the samples in our total RNA-seq datasets and hemisphere-specific clustering of the samples based on the expression patterns of the 13 selected basal ganglia-specific genes in our RNA-seq datasets, which underline the proposition by Biswas et al*.* [32] that regional hemisphere-specific datasets are needed for identifying detailed molecular profiles in specific tissue regions.

As an essential improvement to the BTI approach previously described by Zaccaria et al*.* [12], our method did not make any modifications to the standard surgical DBS procedures, and our approach allowed routine sample collections from the guide tubes from both hemispheres of the patients, without the need for sample pooling for subsequent analyses. Pooling of samples may increase the number of proteins identified, but at the same time, it masks sample-specific proteoforms and post-translational modifications and may lead to the loss of valuable information on individual patients [42]. For their analysis, Zaccaria et al. pooled samples from six patients and both hemispheres for in-gel fractionation and subsequent MS analysis [12]. In general, this sample pooling may increase the number of proteins identified, but it may also lead to the loss of information on sample variation, failure to detect biomarkers and false identification of others [42]. Molinari et al*.* found that pooled samples are not equivalent to the average of biological values and pooling can affect statistical analysis [42]. The pooling of the BTI samples [12] for downstream analyses has led to the loss of substantial patient- and hemisphere-specific information, whereas our datasets are patient- and hemisphere-specific.

In this study, all samples were analyzed separately, and the number of proteins identified varied among the samples, with only 18 proteins common to all proteomics datasets. Missing values (i.e., not every protein that is identified in one sample is detected and quantified in other corresponding samples) are a well-known issue in data-dependent acquisition [43]. In our data, the problem of missing values may be caused by low sample amount, and the high between-sample variability, i.e., the samples had variable amounts of blood, and different brain areas of different patients with different diseases were sampled on different time points. Also, the current data were acquired using a sampling method that was still under development. In future studies, removal of blood from the samples prior to the LC–MS analysis, if managed with minimal sample loss, or alternative techniques, such as data-independent acquisition or quantification using isobaric labeling, could be used to alleviate the missing value problem [44].

Comparison between our datasets and those generated by the BTI approach by Zaccaria et al*.* confirmed that the approach in general is reproducible and robust despite the differences in sample collection procedure and in analysis platforms. Both transcriptomics and proteomics datasets contained substantial number of common identifiers and GO enrichment analysis resulted in similar sets of enriched terms. Also, comparison of our datasets to postmortem datasets [31, 32] focusing on corresponding anatomical regions showed that data obtained by our approach are well aligned with previous knowledge. GO term and KEGG pathway enrichment analysis revealed that all the compared proteomics datasets have similar sets of enriched terms and SynGO analysis of the transcriptomics datasets confirmed the enrichment of synapse-related transcripts.

## Conclusions

Our improved approach can be used to provide novel information on brain tissue-specific transcript variants, proteoforms and post-translational modifications, which are valuable additions to the current knowledge of brain transcriptome and proteome landscapes in vivo. The current method does not require any modifications to standard surgical protocols, and it allows collecting and analyzing fresh, hemisphere-specific samples from individual patients. In the future, as proteomics and transcriptomics techniques are becoming more sensitive and with development of new methods, the approach described here will be a valuable tool with which to access fresh brain-derived material for novel discoveries. Combination of the patient-derived proteomics and transcriptomics data with experiments utilizing patient-derived cells and disease modelling would advance personalized medicine and studies in the field of neurological diseases.

## Supplementary Information

**Additional file 1**. Proteomics dataset, quantified proteins

**Additional file 2**. GO and KEGG pathway enrichment analysis for proteomics data and lists of Uniprot identifiers used for enrichment analysis

**Additional file 3**. SynGO enrichment analysis for RNA-seq data, filtered gene list and enrichment analysis

**Additional file 4**. RNA-seq dataset, feature counts in each analyzed sample

**Additional file 5**. Pilot Western Blotting experiment; results, materials and methods

## Data Availability

The processed RNA-seq and LC–MS datasets are described and available via the BioStudies database (https://www.ebi.ac.uk/biostudies/) under accession number S-BSST667. Raw data from the LC–MS analysis are accessible via Proteomics IDEntifications Database (PRIDE, https://www.ebi.ac.uk/pride/archive/projects/PXD026936) [45, 46]. The raw datasets generated by RNA sequencing during the current study are not publicly available, because of the risk that the data originated by sequencing-based technology may reveal enough variants to identify an individual. All the rest relevant data are supplied within the current publication.

## Abbreviations

BTI: Brain tissue imprint
DBS: Deep brain stimulation
GO: Gene ontology
GPi: Globus pallidus interna
LC–MS: Liquid chromatography–mass spectrometry
MER: Microelectrode registration
MRI: Magnetic resonance image
PD: Parkinson’s disease
RNA-seq: RNA sequencing
STN: Subthalamic nucleus
VIM: Ventral intermediate nucleus of the thalamus
